# The Involvement of Intra-Hippocampal Dopamine Receptors in the Conditioned Place Preference Induced By Orexin Administration into the Rat Ventral Tegmental Area

**Published:** 2019

**Authors:** Farzaneh Sadat Naghavi, Parastoo Namvar, Fatemeh Sadeghzadeh, Abbas Haghparast

**Affiliations:** *Neuroscience Research Center, School of Medicine, Shahid Beheshti University of Medical Sciences, Tehran, Iran.*

**Keywords:** Reward, Dopaminergic system, Orexin, Hippocampus, Ventral tegmental area, Rat.

## Abstract

The activity of dopamine (DA)-containing neurons in the ventral tegmental area (VTA) is a key mechanism in mesolimbic reward processing that has modulatory effects on different diencephalic structures like hippocampus (HIP), and receives inhibitory feedback and excitatory feed forward control. In addition, within the hippocampus, DA receptors are mostly located in the dorsal part (CA1) and dopaminergic innervations are predominant in this sub-region. The current study aimed to examine the effect of intra-hippocampal CA1 administration of SCH23390 and Sulpiride as D1- and D2-like receptor antagonists on the acquisition of orexin-induced conditioned place preference (CPP), respectively. Cannulas were unilaterally implanted into the VTA and HIP of adult male albino Wistar rats weighing 200-250 g. For induction of CPP, orexin A (10 ng/0.3 µL saline) was daily microinjected into the VTA during a three-day conditioning phase. Thereafter, various doses of SCH23390 and Sulpiride (0.25, 1 and 4 µg) were unilaterally injected into the CA1 during this 3-day conditioning phase after intra-VTA administration. The conditioning score was then calculated. Results revealed that intra-CA1 administration of D1- and D2-like receptor antagonists during the 3-day conditioning phase attenuated the acquisition of place preference by orexin A in a dose-dependent manner. It seems the effect of D2-like receptor antagonist within the CA1 region of hippocampus on this phenomenon was found to be more considerable than that of D1-like receptor antagonist. It is concluded that orexin-induced CPP may be mediated, at least in part, by stimulation of DA receptors in the CA1.

## Introduction

Reward can be perceived as stimuli that positively augment behavior, usually inducing a conscious experience of enjoyment (1). Mesolimbic dopaminergic pathway is one major type of dopamine signals in mammals originating from ventral tegmental area (VTA) playing a pivotal role in reward processing (2, 3). The VTA neurons innervate the different cortical and limbic regions like the lateral hypothalamus (LH) and hippocampal complex and receive several inputs from these regions therefore constituting regulatory mechanisms of the reward system (4). Various stimuli could cause an increase in dopamine (DA) release by fluctuating the firing patterns in DA neurons (5). The roles of endogenous orexin neuropeptides in various physiological functions such as opioid effects, reward seeking behavior, neuroendocrine regulation, sleep-wake control, and nociceptive behavior have been previously established (6-8). It has been reported that direct injection of orexin into the VTA induces the conditioned place preference (CPP) in a dose-dependent manner (9). Orexin exerts an activation effect on dopaminergic neurons and non-dopaminergic neurons through orexin receptors which have been found at high density in the VTA (10, 11). As the main catecholamine in the mammalian brain, dopamine is involved in various brain functions such as reward (12) via its receptors, both of which are G-protein coupled and are categorized as two classes nominated as D1-like (D1 and D5) or D2-like (D2, D3 and D4) (13). Both D2-like (14) and D1-like receptor families are found in the hippocampus (HPC) (15). The majority of dopaminergic projections to the HPC originates from the VTA. Dorsal hippocampus (CA1) receives more DA input than other hippocampal sub-regions (16). Furthermore, recent reports support the view that the HPC serves an important role in the process of learning and memory (17) and is associated with addiction to opiates and other drugs. (18). Therefore, the HPC can be considered as a pivotal brain region for reward-related learning functions, such as CPP (18) which is induced by the repeated exposure to drugs and the rewarding effects associated with environmental stimuli (19, 20). It is assumed that learning and memory processes are involved in the CPP procedure (21). It has been reported that the CA1 is known as an imperative hippocampal sub-region associated with the CPP and reward-related learning (19).

The hippocampal CA1 region is suggested to participate in the acquisition and expression of morphine-induced CPP (22). It has also been suggested that dorsal hippocampal lesions caused CPP impairment (19). Evidently, microinjections of D2 antagonists into the CA1 can decrease the expression of intra-VTA morphine-induced CPP, whereas reports of such effects with the D1 receptor have not been consistent in the literature (18, 23). Moreover, the crucial role of D1- and D2-like receptors within the hippocampal CA1 region in the acquisition of intra-VTA morphine-induced CPP has been investigated (18). Previous studies highlighted the rewarding effects of direct orexin administration into the VTA and the involvement of intra-accumbal D1 and D2 receptors in rewarding effects of intra-VTA orexin (9). Nevertheless, there is no evidence to find out the role of D1- and D2-like receptors within hippocampal CA1 in rewarding effects induced by direct orexin administration into the VTA. Thus, in order to address whether dopamine receptors located in the CA1 affect the acquisition of CPP followed by intra-VTA administration of orexin, we tried to examine the effects of intra-CA1 administration of the selective D1- and D2-like receptor antagonists on the acquisition of conditioned place preference by intra-VTA orexin.

## Experimental

*Animal housing*

Experiments were performed on 147 adult male albino Wistar rats (200–250 g from Pasteur Institute, Tehran, Iran) which were housed on a 12 h light/dark cycle at a temperature controlled room and allowed free access to food and water. All methods used were in compliance with guidelines for Care and Use of Laboratory Animals (National Institutes of Health Publication No. 80-23, revised 1996) and were approved by the Research and Ethics Committee of Shahid Beheshti University of Medical Sciences (IR.SBMU.PHNS.REC.1396.126), Tehran, Iran.

*Stereotaxic surgery*

Rats were mounted on a stereotaxic apparatus (Stoelting, USA) under xylazine (10 mg/kg) and ketamine (100 mg/kg) anesthesia. Then, using a stereotaxic apparatus, two guide cannulae were unilaterally implanted 1 mm above the VTA (AP = 4.8 mm caudal to bregma, Lat = ±0.8 mm and DV = 8.3 mm) and CA1 (AP = 3.8 mm posterior to bregma, Lat = ±1.6 mm and DV = 3.6 mm). These coordinates were in accordance with the rat brain atlas of Paxinos and Watson (24). The guide cannulae were then secured in place using screws anchored to the skull and dental acrylic cement. After the surgery, the animals were allowed to recover for 5-7 days. 

*Drugs preparation*

The compounds used in the present study are as follows: Orexin A (Tocris Bioscience, Bristol, UK), and SCH23390 (Tocris Bioscience, Bristol, UK), a D1-like receptor antagonist, dissolved in normal saline; Sulpiride (Tocris Bioscience, Bristol, UK), a D2-like receptor antagonist, dissolved in dimethyl sulfoxide (DMSO) (Sigma–Aldrich, Germany). Control animals received either saline or DMSO. All drugs were freshly prepared on the day of experiment.

*Microinjection procedure*

Microinjections were performed by attaching an injection needle (30-gauge injector cannula) to polyethylene tubing (PE-20), and then, free end of the tubing was attached to a 1-µL Hamilton syringe. An appropriate amount of injection was drawn (CA1: 0.5 µL/rat, VTA: 0.3 µL/rat) into the tubing, and was infused over 60s while the animal roamed freely. 

*Conditioning place preference apparatus*

*Apparatus*

The apparatus contains three-compartment conditioning boxes. The null compartment is considered as a start box (30 × 15 × 40 cm) and usually serves as a connection between two main equal-sized compartments (30 × 30 × 40 cm), and is separated by a removable Plexiglas wall. Conditioning took place in one of two main compartments, which differed in pattern and texture. One compartment had white backgrounds with black vertical stripes and a smooth floor. Another compartment had black horizontal stripes with a rough floor. Before the conditioning session, the animals display no baseline preference for either of these two compartments (25).

*Conditioning place preference protocol*

Conditioned place preference includes three phases: pre-conditioning, conditioning, and post-conditioning. 

Pre-conditioning phase. In the pre-conditioning procedure (day 1) through removal the guillotine door the animals freely access to all compartments. It is conducted to determine the baseline chamber preference and consists of a 10-min trial for each rat. Each animal displacement was recorded. 

Conditioning phase. One day after the pre-conditioning session the conditioning phase consisting of six 30-min sessions (three saline and three drug pairing) in a three-day schedule was started (2^nd^ day-4^th^ day)[38]. These sessions were carried out twice each day and the animals were restricted for 30 min after first injection to one side of the two-sided compartment, removed to their home cages for 6 h, and then subjected to another 30-min conditioning trial. On each day, separate groups of the animals received a conditioning session with drug and another with saline. Treatment compartment and order of presentation of drug/saline were counterbalanced for either group.

Post-conditioning phase. A single CPP assessment session followed the last conditioning session experienced by each rat after 24 h, in a drug free state. In this session, the rats were tested only once, having access to all compartments for a 10-min period. The time spent and distance traveled in each compartment for each rat during a 10-min period were recorded by a 3CCD camera (Panasonic Inc., Japan) and analyzed using the Ethovision software (XT, Version 7), a video tracking system for automation of behavioral experiments (Noldus Information Technology, the Netherlands). The conditioning score, as a preference index, was calculated as the time spent in the drug-paired compartment minus the time spent in saline-paired compartment. The total distance traveled was also recorded in all control and experimental groups.

*Experimental design*

*Effects of intra-CA1 administration of D1-like receptor antagonist, SCH23390, on the acquisition of intra-VTA orexin-induced conditioned place preference*

In order to investigate the role of intra-CA1 D1 receptors in the acquisition of orexin-induced CPP, different doses of SCH23390 (0.25, 1 and 4 µg/0.5 µL DMSO) were unilaterally injected into the CA1, 5 min prior to intra-VTA injection of orexin A (100 ng/0.3 µL saline) which induced CPP during the 3-day conditioning (acquisition) period. The conditioning score and distance traveled (locomotor activity) were calculated during 10 min on post-test days. In the respective control group, saline (0.5 µL/rat) was unilaterally injected into the CA1 instead of SCH23390 during the acquisition period, prior to microinjection of orexin A within the VTA. In the vehicles group, the animals received saline in the VTA (0.3 µL/ rat) and CA1 (0.5 µL/ rat). In another set of experiment, the animals received the highest dose of SCH23390 within the CA1 before injection of saline into the VTA (0.3 µL/ rat) to indicate the effect of injection of SCH23390 alone into the CA1 on CPP scores and locomotion. In anatomical control group (n = 6), the cannulae were placed in adjacent regions to the CA1 and the highest dose of SCH23390 was injected into the neighboring regions as anatomical misplacements, 5 min prior to microinjection of orexin A into the VTA during the 3-day conditioning period.

**Figure 1:**
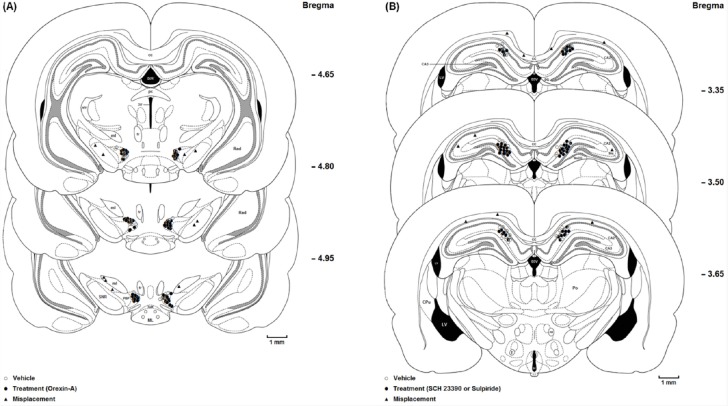
(A) Coronal schematic sections show the microinjection sites in the ventral tegmentum areas (○ Vehicle (DMSO); ● Orexin A microinjection; ▲ Misplacement). D3V: Dorsal 3rd ventricle; cc: Corpus callosum; fr: Fasciculus retroflexus; str: superior thalamic radiation; PC: Paracentral thalamic nucleus; 3V: 3rd ventricle; ml: medial lemniscus; ML: medial mammillary nu, lateral; SuM: Supra mamillary; PBP: Parabrachial pigmented nucleus; SNR: Substantia nigra, reticular part. (B) Coronal schematic sections show the microinjection sites in the hippocampus CA1 (○ Vehicle (saline); ● SCH 23390 or Sulpiride microinjection; ▲ Misplacement). DG: Dentate gyrus; CA2: Field of CA2 of the hippocampus; CA3: Field of CA3 of the hippocampus; MoDG: Molecular layer dentate gyrus; D3V: Dorsal 3rd ventricle; cc, Corpus callosum; LV: Lateral ventricle; 3V: 3rd ventricle; Slu: Stratum lucidum. hyppocampus; Po: Post thalamic nuclear group; f: Fornix; mt: mammillothalamic tract

**Figure 2:**
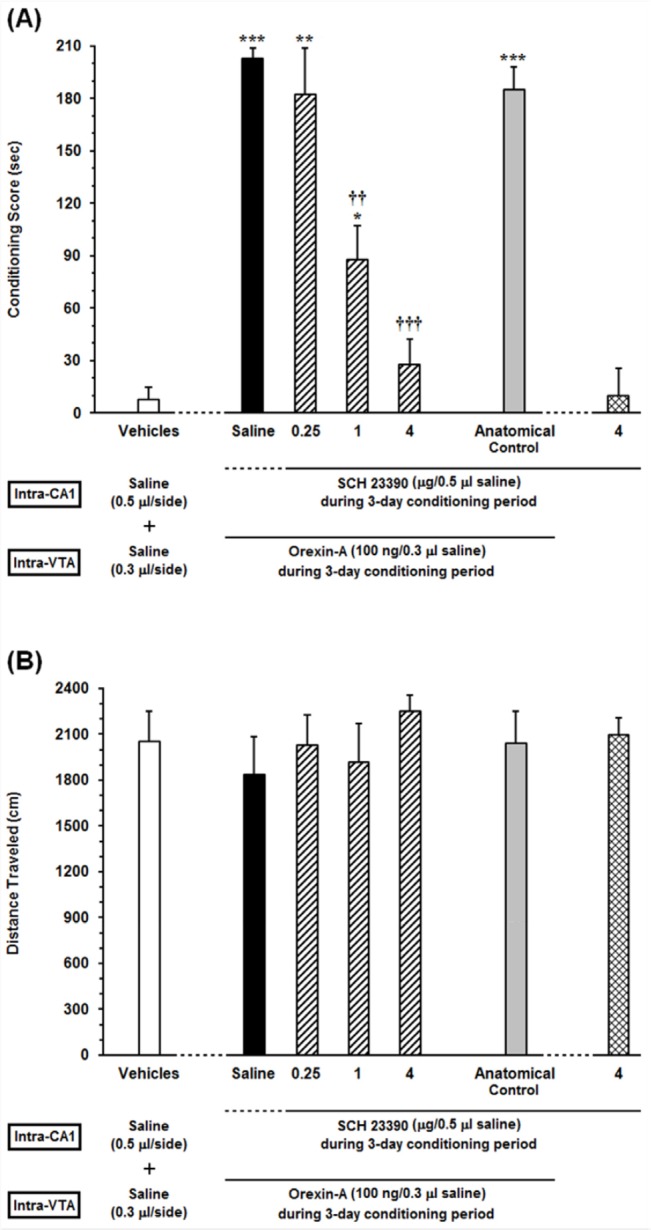
Effects of unilateral microinjection of different doses of SCH23390, a D1-like receptor antagonist, into the dorsal hippocampus (CA1) on intra-VTA orexin-induced CPP (A) conditioning score and (B) locomotor activity induced by microinjection of Orexin into the VTA during 10-min period on post-test day. In this set of experiments, animals received different doses of SCH23390 (0.25, 1 and 4 µg/0.5 µL saline) or Saline (0.5 μL/side) as a vehicle in the CA1, 5 min prior to intra-VTA injection of orexin A (100 ng/0.3 µL saline) during the 3-day conditioning period. The anatomical control group (n = 6) received the highest dose of SCH23390 (4 µg/0.5 µL saline), 5 min prior to intra-VTA orexin-induced conditioned place preference. Each bar shows the mean ± SEM for 6 rats. *P < 0.05, **P < 0.01, ***P < 0.001 different from the vehicle-control group (white bar). ††*P *< 0.01, †††*P *< 0.001 different from the control group (black bar)

**Figure 3:**
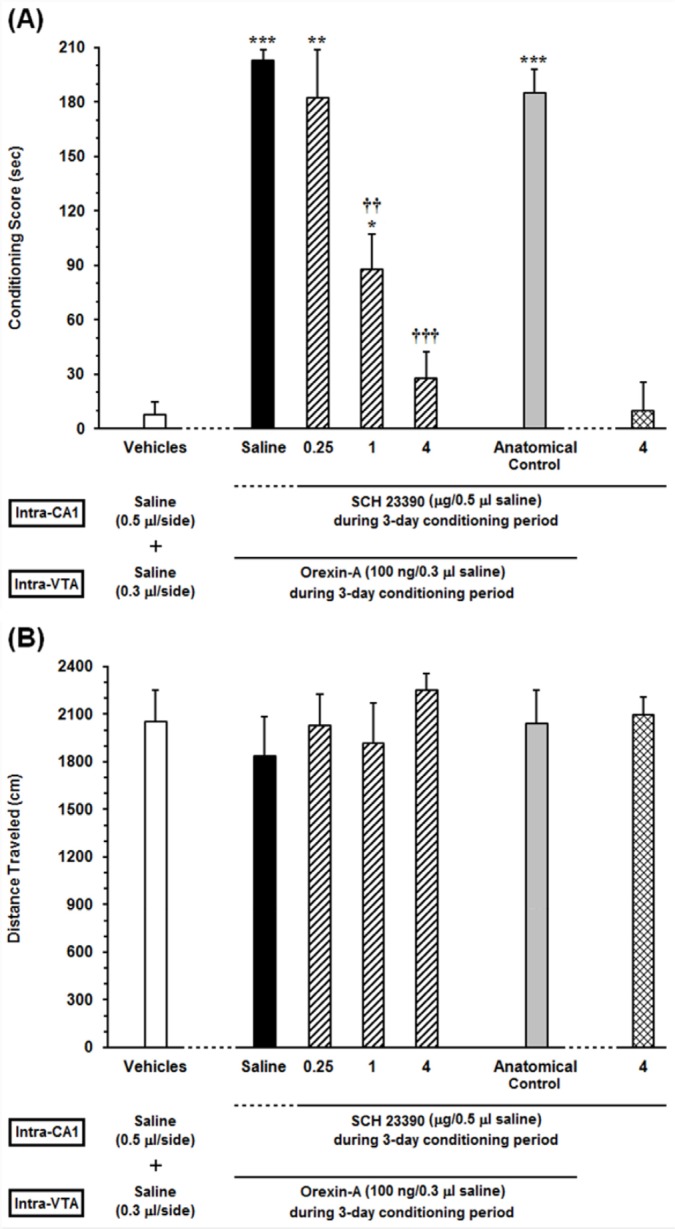
Effects of unilateral microinjection of different doses of Sulpiride as a D2-like receptor antagonist, into the dorsal hippocampus (CA1) on intra-VTA orexin-induced CPP (A) In this set of experiments, animals received different doses of (0.25, 1 and 4 µg/0.5 µL DMSO) or DMSO (0.5 μL/side) as a vehicle in the CA1, 5 min prior to intra-VTA injection of orexin A (100 ng/0.3 µL saline) during the 3-day conditioning period and the conditioning scores were measured in post-conditioning day. (B) Mean locomotor activity of all the groups in this set of experiment during 10-min period on post-test day. The anatomical control group (n = 6) received the highest dose of Sulpiride (4 µg/0.5 µL DMSO), 5 min prior to orexin-induced conditioned place preference. All data are expressed as mean ± SEM for 6-7 rats. *P <0.05, ***P < 0.001 different from the vehicle-control group (white bar). †††*P *< 0.001 different from the control group (black bar)

**Figure 4:**
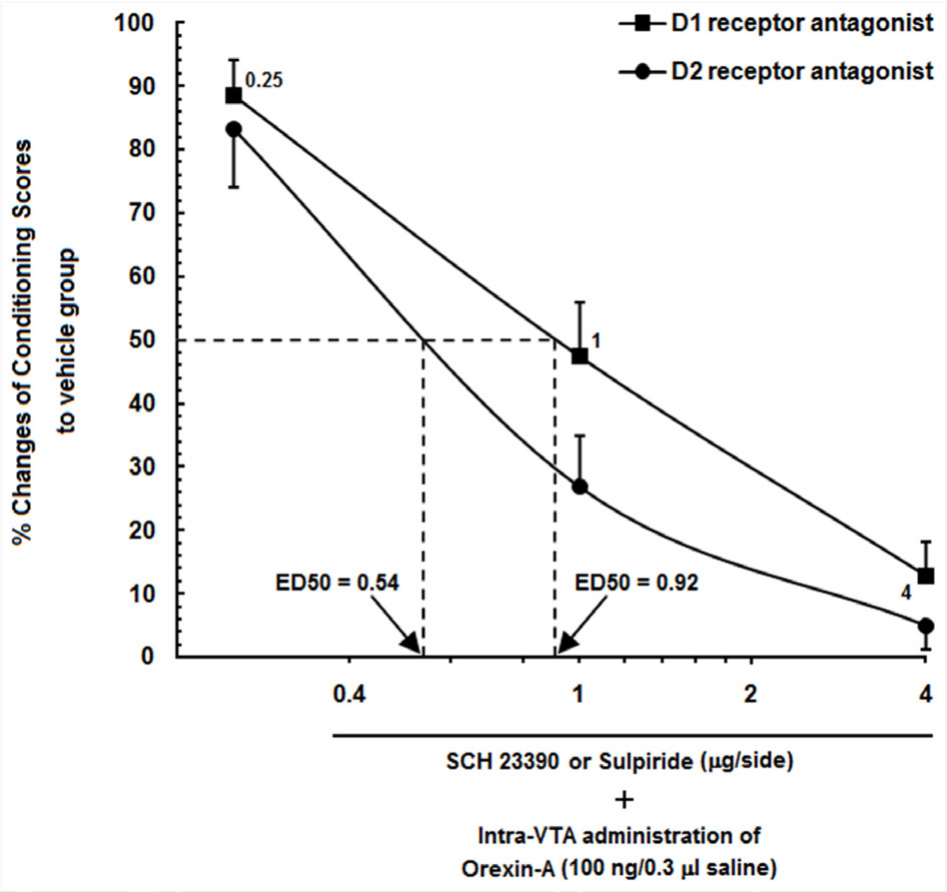
Log conditioning score curve based on the effect of different doses of SCH23390 and Sulpiride (0.25, 1 and 4 µg/0.5 µL saline or DMSO) into the CA1 on the orexin-induced CPP. ●% changes in responses of animals that received different doses of SCH23390 into the CA1. ■ % changes in responses of animals that received different doses of Sulpiride into the CA1

*Effects of intra-CA1 administration of D2-like receptor antagonist, Sulpiride, on the acquisition of intra-VTA orexin-induced conditioned place preference*

To test the possible role of intra-CA1 D2 receptors in intra-VTA orexin-induced CPP, Sulpiride, as a D2 receptor antagonist, was unilaterally microinjected into the CA1 at several doses (0.25, 1 and 4 µg/0.5 µL DMSO), 5 min prior to the microinjection of orexin A into the VTA during the 3-day conditioning (acquisition) period. In the vehicles group, the rats received DMSO (0.5 µL/rat) unilaterally in the CA1 instead of Sulpiride and saline (0.3 µL/rat) instead of orexin A in the VTA during conditioning period. In the respective control group, DMSO (0.5 µL/rat) was unilaterally injected into the CA1 instead of Sulpiride before microinjection of orexin A within VTA, during the acquisition period. In another set of experiment, the animals received the highest effective dose of Sulpiride within the CA1 before injection of saline into the VTA (0.3 µL/rat) to indicate the effect of Sulpiride alone into the CA1 on CPP scores and locomotion. In the anatomical control group (n = 6), the highest dose of Sulpiride was injected in adjacent regions to the CA1 as anatomical misplacement, 5 min prior to the microinjection of orexin A into the VTA during the 3-day conditioning period. A day after the last session conditioning, the conditioning score and distance traveled were calculated during a 10-min period on the post-test day by video tracking system.

*Histological verification*

At the end of the experiments, the rats were deeply anesthetized with ketamine and xylazine and perfused transcardially with a 0.9% saline solution followed by a 10% formalin solution. The brains were removed and stored in 10% formalin. The neuroanatomical location of cannula tips were confirmed using the rat brain atlas (24). The data reported here were only from animals in which the placements of cannula tips were histologically verified in the VTA (Figure 1A) and CA1 (Figure 1B). Ten animals with misplaced cannulae were excluded from further analysis.

*Statistics*

Data are presented as mean ± SEM for each experimental group. To compare the conditioning scores or locomotor activity obtained in all control and experimental groups, one-way analysis of variance (ANOVA) and blocks randomized model followed by post-hoc analysis (Dunnett’s or Newman-Keuls test) were used, as appropriated. Statistical significance was set at *P* < 0.05.

## Results

What we know about effective doses of orexin is largely based upon our previous and current studies reporting that intra-VTA administration of 100 ng/rat orexin A could induce the CPP in such a manner to achieve the best result. Therefore, we used that as the effective dose for continuing the rest of study.

*Effects of intra-CA1 administration of SCH23390, D1-like receptor antagonist, on the acquisition of intra-VTA orexin-induced CPP*

To assess the receptor specific role of D1 receptors in the CA1, the effects of intra-CA1 administration of different doses of SCH23390 (0.25, 1 and 4 µg/0.5 µL saline) as a selective D1-like receptor antagonist on intra-VTA orexin-induced CPP were examined. The results obtained from this experiment are presented in Figure 2. One-way ANOVA followed by Newman-Keuls test [F (6, 43) = 29.35, *P* < 0.0001] indicated significant differences in conditioning scores between the vehicles (saline unilaterally microinjected into the CA1and VTA, respectively) and experimental groups (Figure 2A). It is apparent from the figure that injection of different doses of SCH23390 (0.25, 1 and 4 µg/0.5 µL saline) into the CA1 caused a decrease in the time spent on the drug-paired compartment compared with the time spent on the saline-paired compartment (CPP score) in a dose-dependent manner. However, this reduction is significant at two higher doses of SCH23390 (1 and 4 µg/0.5 µL saline) in comparison with saline-control group. Nevertheless, intra-CA1 microinjection of the highest dose of SCH23390 (4 µg/0.5 µL saline) alone did not induce CPP. On the other hand, the anatomical control group showed that misplace cannula injection produced no effect on the acquisition of intra-VTA orexin-induced CPP. Further statistical analysis demonstrated that intra-CA1 administration of the D1 receptor antagonist had no effect on the locomotor activity during 10 min on post-test day [*F *(6, 43) = 0.4574, *P *= 0.835; Figure 2B]. 

*Effects of intra-CA1 administration of Sulpiride, D2-like receptor antagonist, on the acquisition of intra-VTA orexin-induced CPP*

In order to evaluate the specific response of D2 receptors within the CA1 in the CPP induced by intra-VTA administration of orexin A, different doses of Sulpiride (0.25, 1 and 4 µg/0.5 µL DMSO) were microinjected into the CA1 prior to intra-VTA administration of orexin A during the conditioning period. One-way ANOVA followed by Newman-Keuls multiple comparison test [*F *(6, 40) = 53.3, *P *< 0.0001; Figure 3A] revealed that there were significant differences in conditioning scores between the vehicles (DMSO and saline unilaterally microinjected into the CA1 and VTA, respectively) and experimental groups (Figure 3A). As shown in Figure 3A, there is a clear trend of decrease in conditioning scores of Sulpiride-treated rats in a dose-dependent manner. However, one-way ANOVA followed by Newman-Keuls test revealed that Sulpiride could significantly affect the CPP only at two higher doses (1 and 4 µg/ 0.5 µL DMSO) [*F *(6, 40) = 53.3, *P *< 0.0001]. Moreover, intra-CA1 administration of the highest dose of Sulpiride (4 µg/0.5 µL DMSO) alone failed to induce CPP. On the other hand, misplaced cannula injection produced no influence on the development of intra-VTA orexin induced CPP. Moreover, one-way ANOVA indicated that on the test day, the animals showed no significant differences in locomotor activity during 10 min on post-test day [F (6, 40) = 0.334, *P* = 0.9146; Figure 3B].

The percentage of conditioning scores of the vehicle groups in the orexin-induced CPP groups, which received saline (0.5 µL/side) into the CA1 was set to 100%, and represented the remaining orexin-induced CPP animals, that received different doses of Sulpiride and SCH23390 into the CA1 as % percent changes in their responses. Figure 4 shows a log conditioning score curve based on the effect of different doses of Sulpiride and SCH23390 into the CA1 on the orexin-induced CPP. As can be seen in this figure, 50% effective dose (ED50) value of Sulpiride on the mentioned group (0.92 µg/0.5 µL DMSO) was greater than that in the SCH23390-treated group (0.54 µg/0.5 µL saline). It indicates that a lower dose of Sulpiride as compared to SCH23390had a more imperative inhibitory effect on CPP induced by intra-VTA administration of orexin A. 

## Discussion

The purpose of the present study was to evaluate the involvement of D1- and D2-like dopamine receptors within the CA1 in development of intra-VTA orexin induced CPP. The major findings of this study were as follows: (i) blockade of D1-like receptors within the CA1 attenuated CPP induced by intra-VTA orexin microinjection. (ii) Blockade of intra-hippocampal D2-like receptors reduced intra-VTA orexin induced CPP. (iii) Inhibitory effect of D2-like dopamine receptor antagonist, Sulpiride, was more effective than D1-like dopamine receptor, SCH23390. No doses of both drugs used in this study had effect on the locomotor activity during 10-min period on post-test day, leading us to deduce that the results of our study are not due to alteration in locomotor activity. As reported in a previous study in our lab (9), the most effective dose of intra-VTA orexin A producing CPP, was the highest dose (100 ng/rat). Thus, in this study, the highest dose of orexin was used to induce CPP. In agreement with a previous study in our lab, the present experiment indicated that intra-VTA orexin (100 ng/rat) could lead to the occurrence of CPP in the rats. Orexin neurons have been demonstrated to originate from the lateral hypothalamus and connect to several neuron systems in brain such as dopaminergic neurons in the VTA. Orexin is known to play a critical role in various physiological process including reward-related behaviors and drug-induced CPP, opioid effects, arousal and nociceptive behavior (6-8, 26). A number of studies have found that LH orexinergic projection to the VTA plays a role in morphine or cocaine-induced CPP (26, 27). There is a large volume of published studies describing the activatory role of orexin in the VTA (10, 28). Our study reported that intra-administration of orexin A into the VTA can significantly lead to CPP in the rats. The first part of our findings along with those from others indicates that central administration of orexin A into the VTA serves to provoke the CPP (9). Moreover, these findings correspond to the results of other studies, showing that there is a functional interaction between orexin and mesolimbic dopamine system (29). There is a considerable agreement that mesolimbic system plays a vital role in reward and reinforcement mechanisms (30, 31). It is supposed that reward and reinforcement mechanisms involved in the learning and memory system (21), and HIP serves an important role in learning functions, such as CPP. There is a dopaminergic loop between the VTA and the HIP that may regulate some kind of curcuitry responsible for memory and learning tasks and can be affected by almost all drugs abuse (32). Moreover, hippocampal CA1 region is implicated in the reward-related learning (33). Our results in consistence with those of other recent studies (34) have underlined the view that the CA1 region of hippocampus, is also a part of the neural system contributed to the reward-related learning. 

Our results can lead to the conclusion that intra-VTA orexin-induced CPP depends on dopamine receptors placed in the CA1 region of hippocampus. In 1997, Maldonado and his colleagues demonstrated that the mice with a genetic disruption of the D2 dopamine receptors using CPP paradigm showed no conditioning to morphine (35). Blockade of D1- or D2-like dopamine receptors in the CA1 region of HIP has been suggested to abolish the acquisition and expression of CPP induced by systemic administration of morphine (22). Previous study showed that just D2-like receptors in the CA1 are involved in the expression of intra-VTA morphine induced CPP (23). Another study investigated the pivotal role of intra-CA1 regions of D1 and D2-like receptores in the acquisition of intra-VTA morphine (18). In support of our study, Taslimi *et al.* (2012) clarified the inhibitory effect of intra-accumbal D1 and D2 dopamine receptore antagonists on the acquisition of intra-VTA orexin induced CPP (9). Broadly speaking, the results of previous studies in accordance with our observation regarding the correlation between D1- or D2-like receptors and reward associated behaviors (36).

On the other hand, our findings showed that the blockade of D1- and D2-like receptors in the CA1 inhibited the CPP induced by orexin A. Nevertheless, it appears that the inhibitory effect of D2-like receptor antagonist on intra-VTA orexin-induced CPP is more than the inhibitory effect of D1-like receptor antagonist. It seems that the effect of VTA dopaminergic neuron by orexin on the D2-like receptors is more significant than that of D1 receptors in the CA1; this effect may be due to different potency levels of these two receptors. Moreover, differential effects of D1- and D2-like receptor antagonists may result from potentially different distributions of these receptors in the CA1. Emerging concepts suggest the approximate distribution of D1- and D2-like receptors in the CA1. The density of D2-like receptor in the CA1 is likely to be less than D1-like receptors (37). Also, SCH23390 seems to bind with high efficacy to 5-HT2 receptors and is known to be a high potent agonist at serotonin_2C_ receptors (38). It is therefore possible that the effect of SCH23390 on the development of CPP is mediated by contribution of D1 and 5-HT receptors. Moreover, it is well known that the stimulation of D1-like dopamine receptors activates adenylate cyclase, whereas the activation of D2-like dopamine receptors inhibits adenylate cyclase (39). Consistent with these opposing intra-cellular signal transductions of D1- and D2-like receptors, differential behavioral contribution is produced by blocking of these two dopamine receptor subtypes within the CA1. Altogether, these data suggest a functional interaction between dopamine receptors within the CA1 and reward system, and their crucial roles in reward function and motivation. However, additional behavioral, electrophysiological, and molecular investigations are needed to elucidate the nature of the role of these receptors in the CA1 and their interactions with reward system. Moreover, future research needs to investigate the potential involvement of intra-CA1 dopamine receptors in retrieval memory of orexin induced CPP. Taken together, the main conclusion to be drown from this study is that both D1- and D2-like receptors are of great importance in the development of CPP, and it seems that there is an interaction between dopaminergic and orexinergic systems in these areas in the reward circuit. The dopaminergic system within the CA1 may therefore represent a target for preventing intra-VTA orexin induced CPP. However, there is a need for more investigations to explain the exact mechanisms responsible for this phenomenon.
